# Psychopharmacology of Tobacco and Alcohol Comorbidity: a Review of Current Evidence

**DOI:** 10.1007/s40429-017-0129-z

**Published:** 2017-02-18

**Authors:** Sally Adams

**Affiliations:** 1 Department of Psychology, University of Bath, 10 West, Bath, BA2 7AY UK; 2 UK Centre for Tobacco and Alcohol Studies, Bath, UK

**Keywords:** Alcohol, Nicotine, Comorbidity, Psychopharmacology, Cross-reinforcement, Cross-tolerance

## Abstract

**Purpose of the Review:**

Comorbidity of alcohol and tobacco use is highly prevalent and may exacerbate the health effects of either substance alone. However, the mechanisms underlying this comorbidity are not well understood. This review will examine the evidence for shared neurobiological mechanisms of alcohol and nicotine comorbidity and experimental studies of the behavioural consequences of these interactions.

**Recent Findings:**

Studies examining the shared neurobiology of alcohol and nicotine have identified two main mechanisms of comorbidity: (1) cross-reinforcement via the mesolimbic dopamine pathway and (2) cross-tolerance via shared genetic and nAChR interaction. Animal and human psychopharmacological studies demonstrate support for these two mechanisms of comorbidity.

**Summary:**

Human behavioural studies indicate that (1) alcohol and tobacco potentiate each other’s rewarding effects and (2) nicotine reduces the sedative and intoxication effects of alcohol. Together, these findings provide a strong evidence base to support the role of the cross-reinforcement and cross-tolerance as mechanisms underlying the comorbidity of alcohol and tobacco use. Methodological concerns in the literature and recommendations for future studies are discussed alongside implications for treatment of comorbid alcohol and tobacco use.

## Introduction

Alcohol and tobacco use independently represent major public health problems, associated with preventable disease and death. Comorbid use of alcohol and tobacco may exacerbate the health effects of either substance alone, with comorbidity associated with increased risk for some types of cancer, e.g. mouth and throat [1–3] and liver cancer [4, 5]. Additionally, for alcohol-dependent individuals, comorbid use increases the risk of tobacco-related diseases (e.g. heart disease and lung disease) [6] and death from tobacco-related complications [7].

Alcohol and tobacco use are highly comorbid [8] and there is a strong correlation between alcohol and nicotine dependency. Alcohol-dependent individuals are three times more likely to smoke than the general population, and individuals who are dependent on tobacco are four times more likely to be alcohol dependent [9]. Furthermore, alcohol-dependent smokers have more severe nicotine dependency and experience greater difficulty quitting than non-alcohol-dependent smokers [10]. Consequently, treatment for drug dependency has faced the challenge of tackling comorbid addiction, with uncertainty regarding whether to treat both substance problems together or separately. Typically, the primary addiction is treated first. However, tobacco dependency is rarely treated in alcohol-dependent individuals as treatment providers view quitting both substances at the same time too difficult and suggest that tobacco treatment may negatively impact interventions for alcohol dependency.

## Psychopharmacology of Comorbidity

Whilst comorbidity of alcohol and tobacco use is clearly evident, the mechanisms underlying this association and consequences for treatment are not well understood. Psychopharmacological studies represent an opportunity for improving our understanding of alcohol and tobacco comorbidity by examining the shared neurobiological mechanisms and the subsequent behavioural effects of these drugs. Examining the interaction between concomitant alcohol and nicotine use and dependency is a major challenge for researchers as these substances have many shared neural mechanisms, making it difficult to tease the behavioural effects of each drug apart. Additionally, the comorbid effects of alcohol and nicotine are difficult to study given the influence of individual differences in age and gender and differential physiological and behavioural effects dependent on the amount of the drug consumed [11, 12]. In this review, we will examine the evidence for shared neurobiological mechanisms of alcohol and nicotine comorbidity and experimental studies of the behavioural consequences of these interactions. Examination of these specific shared mechanisms will contribute to our understanding of the high comorbidity between alcohol and nicotine use and dependency and have important implications for comorbid treatment.

## Neurobiological Mechanisms of Alcohol and Tobacco Use and Dependency

Initiation and experimentation with alcohol and nicotine is largely due to the acute pharmacological effects of these drugs. Nicotine is a stimulant, which can increase alertness and improve concentration, whereas alcohol is a depressant drug with acute effects ranging from increased mood and relaxation to decreased inhibitory control, loss of motor control and reduced reaction times. Acutely, nicotine and alcohol also show interactional effects; e.g. nicotine reduces the sedative effects of alcohol [13] and alcohol potentiates the rewarding effects of nicotine [14]. However, the neurobiological mechanisms of the acute effects for nicotine and alcohol differ. Nicotine primarily acts on the brain via binding and activation of the nicotinic acetylcholine receptor (nAChR). However, alcohol does not bind to one receptor in particular, with activation of many different receptors of several neurotransmitters, e.g. serotonin, dopamine and gamma-aminobutyric acid. Despite these differences in mechanisms of acute effects, there is a growing body of evidence for shared neurobiological mechanisms that underlie the comorbid use of and dependency on alcohol and nicotine.

Studies examining the shared neurobiology and subsequent behavioural effects of alcohol and nicotine have identified two main mechanisms: (1) cross-reinforcement via the mesolimbic dopamine pathway and (2) cross-tolerance via shared genetic and nAChR interaction. Shared psychosocial factors have also been highlighted as a mechanism underlying comorbid use; however these will not be discussed here given the focus of the review on psychopharmacological mechanisms of comorbidity. The following sections will discuss the neural pathways and supporting behavioural evidence for these two mechanisms, evaluating their contribution to our understanding of alcohol and nicotine comorbidity.

## Cross-Reinforcement

Cross-reinforcement refers to the ability of alcohol and nicotine to enhance the motivation to consume the other drug by acting on shared neurobiological mechanisms that underlie the reinforcement of drug effects. Interaction between the reinforcing effects of alcohol and nicotine may occur during acute comorbid use (e.g. intoxication) or may manifest in changes in neurobiological function following repeated use of one or both drugs [15]. Alcohol and nicotine share a reward activation pathway, where both drugs potentiate the rewarding effects of each other via activation of the mesolimbic dopamine pathway. The mesolimbic neural pathway originates in dopaminergic neurons in the ventral tegmental area (VTA) that project and terminate in the nucleus accumbens (NAcc) in the ventral striatum. The experience of reward and reward-seeking is accompanied by release of dopamine in the mesolimbic pathway for natural rewards, e.g. food and sex and drug rewards [16–19]. Specifically, alcohol and nicotine increase dopaminergic neuron firing [20–22] and dopamine release in the mesolimbic pathway [23–25]. Nicotine may activate the mesolimbic pathway via nicotinic acetylcholine receptors (nAChRs) that stimulate VTA neurons to release dopamine in regions including the NAcc [26–30]. The contribution of nAChRs in the reinforcing effects of nicotine is supported by evidence of reduced nicotine administration following blockade of nicotinic receptors in the VTA [31]. Alcohol may influence the mesolimbic pathway by a number of mechanisms. Firstly, alcohol’s interaction with nAChRs may underlie the reinforcing properties of alcohol [32, 33] where nicotine receptor antagonists are shown to increase voluntary alcohol consumption [34]. Secondly, self-administration of alcohol leads to extracellular dopamine release in the NAcc, consistent with a role for mesolimbic dopamine in alcohol reinforcement [35–37]. In addition, alcohol has been shown to change synaptic plasticity in the mesolimbic pathway via dopaminergic mechanisms that may also underlie the development of alcohol reinforcement [38]. Supporting studies have shown that injections of dopamine-releasing agents in the NAcc increases alcohol consumption, whilst injection of agents that reduce dopamine release into the VTA decreases alcohol intake [39].

Research has also examined the role of the mesolimbic pathway to understand the cross-reinforcement between nicotine and alcohol. Evidence for the role of mesolimbic dopamine in interface between alcohol and nicotine has shown the pharmacological blockade of nAChRs in the VTA reduces alcohol intake, indicating that the rewarding effects of alcohol may be dependent on nicotinic receptors [40]. Additionally, microdialysis studies have lent support to the notion that alcohol and nicotine act synergistically on behaviour via dopamine release in the NAcc [41]. Tizabi and colleagues [24, 25] have provided evidence to indicate that co-administration of alcohol and nicotine produces an additive release of mesolimbic dopamine in the NAcc via injections of both drugs into the VTA. Together, these studies suggest that mesolimbic dopamine activity is an important mechanism contributing to the cross-reinforcement and subsequent comorbid use of nicotine and alcohol.

### Animal Studies of Cross-Reinforcement

Animal and human studies have provided robust evidence for the behavioural cross-reinforcement of alcohol and nicotine in support of a shared mesolimbic dopaminergic reward pathway. Studies testing an animal model of cross-reinforcement have consistently demonstrated an interaction between the reinforcing effects of nicotine and alcohol. One line of research has assessed the effects of nicotine on alcohol administration using surgically implanted nicotine releasing capsules or daily injections of nicotine to chronically deliver nicotine. These studies have shown that nicotine increases alcohol self-administration [33, 42–44] and motivation to work for/obtain alcohol [45, 46]. Another line of work has used a relapse/reinstatement animal model to test nicotine’s reinforcement effect on alcohol. Lệ and colleagues [47] trained rats to self-administer alcohol via a lever press. Once this behaviour was established, it was extinguished via the lever press no longer delivering alcohol. Using nicotine injections, Lệ and colleagues [47] were able to reinstate lever pressing in a dose-dependent fashion, indicating that nicotine influences neural pathways underlying alcohol seeking. Few animal studies have investigated the reinforcing effects of alcohol on nicotine.

### Human Studies of Cross-Reinforcement

Human studies are consistent in their support for cross-reinforcement of nicotine and alcohol. However, in contrast to animal studies, the majority of human research has focused on evidence for alcohol increasing the reinforcing properties of nicotine. A small body of research has examined alcohol’s capability to increase urge to smoke and cigarette craving as a measure of cross-reinforcement. One study [48] demonstrated that alcohol (0.4, 0.8 g/kg) increased the urge to smoke in nicotine deprived, heavy drinking, light smokers in a dose-dependent fashion. Smoking urge increases were evident during ascending and descending blood alcohol concentration (BAC) and were greater for positive reinforcing effects than negative reinforcing effects. Similarly, Glautier and colleagues [49] examined the subjective reinforcing effects of alcohol on nicotine, and also included a behavioural measure of cigarette use. This addition is important as subjective cigarette craving (as assessed in King and Epstein [48]) does not necessarily confer to actual smoking choice. By measuring the effects of alcohol (0.5 g/kg) on subjective effects of nicotine and smoking typography Glauteir and colleagues [49] indicated that alcohol increased satisfaction from smoking, length of time spent smoking and number of puffs taken from a cigarette. Together, these studies support a pharmacological priming mechanism of cross-reinforcement, where alcohol increases the subjective reinforcing effects of nicotine.

Further research has examined the behavioural effects of alcohol’s capability to increase the rewarding effects of nicotine and to increase nicotine intake. Early studies of alcohol’s reinforcing effects on nicotine intake demonstrate that alcohol increases the amount, rate and puff volume of cigarette smoking [50, 51]. Similarly, early studies by Mello and colleagues [52, 53] indicate that chronic alcohol administration (15–21 days) increases cigarette use in moderate-heavy smokers However, these studies are significantly limited by small sample sizes and in the case of the majority of studies [50–52] entirely male populations. A more recent study has consolidated these findings using a larger sample size of social drinkers and smokers. Mitchell and colleagues [54] examined the dose-dependent effects of alcohol (0.2, 0.4, 0.8 g/kg) vs. placebo on amount of cigarettes smoked and temporal smoking pattern. Findings indicated that in the hour following a moderate (0.4 g/kg) or higher (0.8 g/kg) dose of alcohol, participants smoked more cigarettes. However, this effect did not extend beyond 1 h suggesting that the cross-reinforcement of alcohol on nicotine may be short lived and restricted to the ascending limb of the blood alcohol curve. This result is in contrast to evidence of alcohol’s potentiation of the urge to smoke on the ascending and descending limb of BAC [48].

Another body of human research has studied the ability of nicotine to increase the reinforcing value of alcohol. Of these studies, several have used a nicotine vs. placebo design to measure the effects of nicotine administration on the rewarding effects of alcohol. Barrett and colleagues [55] used a nicotine (1.2 mg) vs. placebo design to examine performance on a high demand, progressive ratio task that rewarded participants with the opportunity to self-administer alcohol. Findings showed that nicotine led to increased motivation to work for alcohol and increased alcohol consumption. Acheson and colleagues [56•] examined nicotine’s (7, 14 mg) reinforcing effects on subjective ratings of alcohol and alcohol consumption. Nicotine (14 mg) increased alcohol consumption for males, but decreased alcohol consumption for females. Males also reported increased arousal following nicotine pre-treatment, whereas females reported a decrease in positive affect. These findings highlight the importance of studying the effects of comorbidity in male and female drug users as the effects of concurrent use of alcohol and tobacco may differentiate by sex or factors associated with sex (e.g. body mass index) (Table 1).

**Table 1:** Human studies of cross-reinforcement and cross-tolerance

Author (Y\year)	Psychopharmacological mechanism of comorbidity	Sample total, *N* (% males)	Age, years (SD)	Psychopharmacology manipulation	Outcome measure	Results
King and Epstein (2005)	Cross-reinforcement	16 heavy social drinkers, light smokers (69%)	26 (1)	Alcohol (0.4 g/kg, 0.8 g/kg) vs. placebo	Brief Questionnaire of Smoking Urges (BQSU)	Alcohol increased urge to smoke
Glautier et al. (1996)	Cross-reinforcement	16 social drinkers who smoked (NI)	28 (NI)	Alcohol (0.5 g/kg) vs. placebo	Subjective effects of nicotine, smoking topography, exhaled carbon monoxide	Alcohol increased smoking satisfaction, length of time spent smoking, number of puffs taken, amount of tobacco burnt
Griffiths et al. (1976)	Cross-reinforcement	5 alcohol-dependent drinkers (100%)	43 (NI)	Alcohol (12 × 11.14 g ethanol) vs. placebo	Smoking rate	Alcohol increased smoking rate
Mintz et al. (1985)	Cross-reinforcement	14 substance dependent patients (100%)	41 (NI)	Alcohol 0.6 ml/kg vs. placebo	Smoking topography	Alcohol increased amount, puff volume and rate of smoking
Mello et al. (1980)	Cross-reinforcement	6 concurrent users of alcohol and tobacco (100%)	25 (NI)	Alcohol (unrestricted availability for 15 days)	Cigarette use	Alcohol increased cigarette smoking
Mello et al. (1987)	Cross-reinforcement	24 concurrent users of alcohol and tobacco (0%)	26 (1)	Alcohol (unrestricted availability for 21 days)	Cigarette use	Alcohol increased cigarette smoking
Rose et al. (2004)	Cross-reinforcement/cross-tolerance	48 regular drinking smokers (42%)	32 (9)	Ad lib nicotine vs. placebo (alcohol (0.5 g/kg) vs. placebo	Cigarette evaluation questionnaire, smoking withdrawal symptoms, alcohol effect rating.	Alcohol increased smoking satisfaction, liking, calming effects, relief of cigarette craving. Nicotine reversed the sedative effects of alcohol
Mitchell et al. (1995)	Cross-reinforcement	7 social drinking, moderate to heavy smokers (57%)	9.1 (5)	Alcohol (0.2 g/kg, 0.4 g/kg, 0.8 g/kg) vs. placebo	Number of cigarettes smoked, temporal smoking pattern, exhaled carbon monoxide, breathe alcohol level (BAL)	Alcohol increased cigarette consumption on the ascending limb of the BAL only
Barret et al. (2006)	Cross-reinforcement	15 occasional smokers (100%)	22 (2)	4 nicotine-containing cigarettes (1.2 mg) vs. 4 denicotinised cigarettes (placebo)	Progressive ratio (PR) task (alcohol vs. placebo rewards), alcohol self-administration	Nicotine increased PR for alcohol and alcohol self-administration
Acheson et al. (2006)	Cross-reinforcement	34 light smoking, social drinkers (65%)	Males 27 (5) Females 23 (2)	Transdermal nicotine patch (7 mg, 14 mg) vs. placebo	Subjective effects of alcohol, alcohol self-administration	Nicotine increased alcohol consumption and arousal for males. Nicotine decreases alcohol consumption for females and positive affect
Kouri et al. (2004)	Cross-reinforcement	12 occasional drinking smokers (100%)	28 (NI)	Transdermal nicotine patch (21 mg) vs. placebo, alcohol 0.4 g/kg, 0.7 g/kg)	Subjective effects of alcohol, physiological effects of alcohol	Nicotine increases alcohol intoxication, wanting to drink more and alcohol-induced heart rate
Oliver et al. (2013)	Cross-reinforcement/cross-tolerance	87 current smokers and drinkers (67%)	29 (9)	1 nicotine-containing cigarette (0.6 mg) vs. 1 negligible nicotine cigarette (0.05 mg) (placebo), alcohol (0.3 g/kg males, 0.27 g/kg females) vs. placebo	Brief Questionnaire of Smoking Urges (BQSU), Alcohol Urges Questionnaire (AUQ)	Combined nicotine and alcohol increased cigarette craving, and alcohol craving for females only. Alcohol blocked the satiating effect of nicotine on cigarette craving
Perkins et al. (1995)	Cross-reinforcement/cross-tolerance	18 smokers, moderate drinkers (50%)	22 (1)	Nicotine nasal spray (20 μg/kg) vs. placebo, alcohol (0.5 g/kg) vs. placebo	Subjective drug effects, cardiovascular response	Combined alcohol and nicotine increases subjective ratings of head-rush, intoxication, arousal and cardiovascular response. Nicotine reduced increases in fatigue and intoxication and decreases in arousal due to alcohol

*NI* not indicated)

Recent work has sought to extend the work of studies that examine the effects of either alcohol or nicotine on the other drug, by examining concurrent administration of alcohol and nicotine. Rose and colleagues [14] examined the effects of alcohol (0.5 g/kg) vs. placebo and an ad lib smoking period on subjective drug effects including stimulation and satisfaction and relief of craving. Results indicated that alcohol potentiated the positive rewarding effects of nicotine as indexed by increased self-report smoking satisfaction, enhanced stimulant and calming effects of nicotine and increased relief of cigarette craving. In addition, nicotine reversed the sedative effects of alcohol (this finding will be discussed further in the cross-tolerance section). A further study by Kouri and colleagues [57] examined the effects of nicotine via transdermal patch on the subjective and physiological effects of either a moderate (0.4 g/kg) or high (0.7 g/kg) dose of alcohol. Results indicated that nicotine pre-treatment increased feeling drunk, the effects of alcohol and wanting to drink more. Furthermore, alcohol-induced increased heart rate was enhanced following nicotine pre-treatment. Consistent with the findings of Mitchell and colleagues [54], these effects were most pronounced in the first hour following alcohol consumption and diminished after 2 h. A further study [58•] examined the separate and combined administration of nicotine and alcohol on craving for each drug using a double-bind study of alcohol (0.30 g/kg males, 0.27 g/kg females) vs. placebo and nicotine (0.6 mg) vs. placebo. Findings indicated that combined administration of nicotine and alcohol increased cigarette craving for all participants and alcohol craving for females and light drinkers only. A similar study by Perkins and colleagues [59] examined the effects of concurrently administered nicotine (20 μg/kg) and alcohol (0.5 g/kg) on subjective drug effects, mood and cardiovascular response. Results showed that combined administration of alcohol and nicotine increased head-rush, intoxication, arousal cardiovascular measures. These findings suggest had an additive effect of both drugs on pharmacological measure of drug reinforcement. Together, this study with other discussed here demonstrates a role for behavioural cross-reinforcement in the comorbid use of alcohol and tobacco. The findings of these studies also lend support for the role of the mesolimbic dopaminergic pathways in the comorbidity of nicotine and alcohol use.

## Cross-Tolerance

Tolerance is a process demonstrated following repeated drug use, where by continued use of a fixed amount of a substance produces a lesser effect (e.g. euphoria, buzz). Therefore, a greater amount of the drug is required to achieve the same, initial effect. The development of tolerance is thought to be instrumental in the escalation of drug-intake and progression to drug dependence. Repeated use of both alcohol and nicotine use can facilitate tolerance to the drugs’ pharmacological effects [60, 61]. In addition to tolerance to each individual substance, pharmacological interactions between alcohol and nicotine are evident in the reduction of response to one drug via use of the other. Development of cross-tolerance may contribute to the comorbid use of nicotine and alcohol use via a mechanism of genetic predisposition [62]. Evidence has demonstrated that individuals with a family history of alcohol dependency may inherit a diminished sensitivity to the pharmacological effects of alcohol, including less intoxication and body sway [63]. Similarly, Health and Colleagues [64] reported evidence of a role for genetic predisposition in sensitivity to alcohol’s effects in individuals with no history of familiar alcohol dependency. Together, these studies suggest that decreased reactivity to alcohol may lead to increased and heavy alcohol use due to reduced experience of the pharmacological effects of alcohol. Further research, has also indicated that current smokers report a diminished intoxicating effect of alcohol, compared to former and non-smokers [64] and for female smokers a genetic association between smoking status and alcohol intoxication [65]. At present, there is limited knowledge of the precise genes involved in the effects of nicotine on diminished response to alcohol intoxication. However, these studies do indicate cross-tolerant effects between nicotine and alcohol that reflects a reduced response to alcohol in those who smoke.

Another line of research has focused on nicotinic receptors as a possible mechanism underlying the cross-tolerance of nicotine and alcohol. Evidence has demonstrated that alcohol enhances and inhibits the function of several nAChR subtypes [66–68]. Through mechanisms of both enhancement and inhibition of nAChR subtypes, alcohol is able to affect transmission at these receptors and nicotine-induced signalling [69]. Several studies have demonstrated that modulation of these receptors can alter the behavioural and neurotoxicological effects of alcohol [33, 69, 70], indicating that shared nAChR sites may contribute the comorbid use of alcohol and tobacco. A recent study indicated the role of nAChRs in the cross-tolerant effects of both drugs. Taslim and colleagues [71] showed that nicotine reduced alcohol-induced incoordination via nAChR subtype function, suggesting a role for nAChR subtypes *α*4*β*2 and *α*7 in the behavioural cross-tolerance of nicotine and alcohol.

### Animal Studies of Cross-Tolerance

Cross-tolerance of alcohol and nicotine is a complex process to disentangle and evaluate given the repeated comorbid use of both drugs in humans. However, animal studies of cross-tolerance have demonstrated that reduction of response to nicotine/alcohol via repeated exposure to the other drug is an important motivating factor in comorbid use. These studies have examined cross-tolerance between nicotine and alcohol on numerous measures of the pharmacological effects of both drugs, including changes in temperature and motor activity. One body of research has assessed the effects of repeated alcohol exposure on cross-tolerance to the effects of nicotine in rodents. One study [72] demonstrated that chronic alcohol administration (via a liquid diet) initiated tolerance for alcohol’s behavioural effects. Additionally, chronic alcohol exposure induced cross-tolerance for nicotine. A further supporting study [73] of 4-day alcohol administration in adolescent mice demonstrated tolerance to alcohol’s hypothermic response (i.e. reduced temperature). At 30 days post alcohol administration, female mice were cross-tolerant to nicotine’s effects on temperature and locomotor activity (i.e. location exploration). These studies support the notion that alcohol and nicotine share neurobiological sites of action.

Another group of animal studies have examined the effects of repeated nicotine administration on cross-tolerance of alcohol’s effects. A series of studies by Parnell, Chen and Colleagues [74–76] have demonstrated that administration of a range of nicotine doses (0.25, 0.5, 1, 2, 4, 6 mg/kg), reduces peak BAC following a dose of alcohol, indicative of cross-tolerant effect of nicotine on alcohol. Further evidence of nicotine’s cross-tolerant effects has been provided by studies of repeated nicotine infusion in selectively bred mice. Research has shown that inbred DBA/2 mice exposed to intravenous infusion of nicotine (0.25–0.8 mg/kg/h) for 10–14 days developed tolerance for several effects of nicotine and cross-tolerance to the hypothermic effects of alcohol [77, 78]. These data support the genetic basis of the cross-tolerance of nicotine and tobacco, reflecting a reduced response to alcohol following repeated nicotine exposure.

### Human Studies of Cross-Tolerance

Compared with human studies of cross-reinforcement, fewer studies have produced translational evidence of cross-tolerant effects of nicotine and alcohol in humans. However, of the studies that did examine this research question, most have examined cross-tolerance as a mechanism mitigating the aversive effects of one drug on the other via measures of intoxication, craving and drug use. Several studies have investigated the combined effects of alcohol and nicotine administration on the subjective and behavioural interaction of these drugs. A study by Oliver and colleagues [58•] demonstrated that co-administration of a low-dose alcohol (0.30 g/kg males, 0.27 g/kg females) blocked the satiating effect of nicotine on cravings to smoke. Similarly, Rose and colleagues [14] examined the effects of alcohol (0.5 g/kg) and an ad lib smoking period on subjective drug effects. Results demonstrated that nicotine reduced the sedative effects of alcohol. A third study by Perkins and colleagues [59] examined the combined effects of alcohol (0.5 g/kg) and nicotine (20 μg/kg) administration on subjective drug effects. Findings indicated that nicotine reduced the intoxicating effects of alcohol and eliminated alcohol’s sedative effects during descending BAC. Together, the findings of these studies suggest a mechanism of alcohol and nicotine cross-tolerance whereby nicotine reduces the subjective intoxication and sedative effects of alcohol.

## Conclusions

Studies examining the behavioural effects of alcohol contribute to our understanding of concurrent alcohol and tobacco use in several important ways. Findings of behavioural studies discussed here indicate the role of two key psychopharmacological mechanisms in the comorbid use of alcohol and tobacco; cross-reinforcement and cross-tolerance. Studies of cross-reinforcement indicate that alcohol and nicotine potentiate each other’s rewarding effects, as evidenced by increased craving, subjective rewarding effects, consumption and motivation to work for the other drug. Findings of these studies indicate that the comorbidity of alcohol and tobacco use is in part driven by an interaction between the reinforcing effects of alcohol and nicotine on enhanced motivation to consume the other drug. The studies reviewed here also indicate that alcohol and nicotine have a cross-tolerant effect, where evidence demonstrates that nicotine reduces or blocks the sedative and intoxication effects of alcohol. Nicotine’s attenuation of these effects may serve to eliminate some the negative effects of alcohol that limit alcohol consumption, e.g. tiredness and drunkenness. Therefore, these findings suggest that the cross-tolerance effect of nicotine on alcohol’s sedative effects is a potential mechanism underlying comorbid use of alcohol and nicotine.

The findings discussed here have clinical implications for treatment of comorbid alcohol and tobacco use. Until recently, smoking and drinking were viewed as separate targets in interventions aimed at reducing tobacco and alcohol use. Similarly, alcohol and tobacco dependence were approached separately or individually in treatment, with alcohol dependency often being treated first, in isolation. Drobes [79] suggests that smoking cessation programmes were deemed as contradictory to treatment of alcohol dependency, where tobacco dependency was regarded as a more trivial problem compared with alcohol misuse. Furthermore, treatment of tobacco dependency was considered to have a negative impact on treatment outcomes for treatment of alcohol dependency [80]. However, the research examined in this review indicates that combined treatment of comorbid alcohol and tobacco use may lead to more favourable treatment outcomes. Evidence from behavioural studies of cross-reinforcement and cross-tolerance of alcohol and tobacco is potentially useful in identifying individuals at risk for developing heavy comorbid use of alcohol and tobacco and comorbid dependency. Furthermore, understanding the mechanisms underlying alcohol and tobacco’s comorbid use will improve interventions for reducing concurrent alcohol and tobacco use. Studies of cross-tolerance [14, 58•, 59] suggest that nicotine’s reduction of alcohol’s intoxicating and sedative effects may increase the likelihood of greater alcohol use in smokers. This finding has implications for smokers who are looking to cut down or reduce their drinking, where tobacco use may play a role in maintaining drinking behaviours. Smokers and/or health professionals treating them should be aware of the effects nicotine has on the intoxicating effects of alcohol and of the potential of smoking to undermine attempts to reduce drinking due to cross-tolerance effects of alcohol and tobacco. Evidence of alcohol’s cross-reinforcing effect on nicotine also aids our understanding of the role of alcohol effects on nicotine reward in smoking relapse. Research indicates that rates of successful smoking cessation are reduced in smokers with current or past alcohol problems [81], suggesting that co-current tobacco and alcohol use hampers smoking cessation. This finding is supported by evidence from behavioural studies of cross-reinforcement, indicating alcohol’s potentiating effect of nicotine’s rewarding effects, including alcohol’s ability to increase smoking satisfaction [48] and cigarette use [54]. Together, these findings suggest that smokers who are trying to quit should reduce their alcohol consumption or avoid drinking. Evidence from studies of cross-reinforcement also suggest that craving for cigarettes is increased following alcohol consumption [48] and that smokers should also be aware of increased cigarette cravings when drinking alcohol during smoking cessation. Indeed, strategies for reducing alcohol consumption may help attenuate cigarette craving and smoking during smoking cessation.

Interpretation of the discussed findings from behavioural studies, however, should be considered in light of the following methodological limitations. Many of the discussed studies are limited by small sample sizes, with the smallest study having an *n* = 5 [50] and the largest study having a *n* = 78 [58]. It is therefore important that further research using adequately powered studies is conducted to confirm the results of studies with smaller sample sizes. Many of the discussed behavioural studies were also conducted with only male participants [50–52, 55, 57]. Studies of alcohol and nicotine administration in male and female participants have suggested sex differences in the cross-reinforcement and cross-tolerance of alcohol and nicotine. For example, Acheson and colleagues [56•] found that nicotine increased alcohol consumption in males, but decreased alcohol consumption in females. Sex differences were also reported in the subjective effects of nicotine on alcohol use. Several explanations for these differences in males and females have been suggested, including body composition and social factors [56•]. However, the underlying reasons for sex differences in the pharmacological effects of alcohol and tobacco have not yet been fully explored and therefore warrant further investigation. Another methodological consideration of behavioural studies is the discrimination of alcohol’s effects during ascending and descending BAC. Several studies point towards differential effects of alcohol on nicotine’s rewarding effects at different levels of BAC [48, 54, 58•]. Perkins and colleagues [13] also outline several further methodological problems in interpreting the studies discussed here, including difficulty in controlling nicotine dosing if tobacco/cigarette smoking is the method of administration for nicotine. Studies have begun to use methods of nicotine delivery other than ad lib cigarette smoking, or self-administration of tobacco to eliminate this problem, such as transdermal nicotine patch [57] and nicotine nasal spray [59]. However, further research is required to determine the influence of different methods of nicotine administration. Studies are also limited in their consideration of past history of drug use of study participants [13]. Future behavioural studies of cross-reinforcement and cross-tolerance should seek to examine the effects of nicotine and alcohol co-administration in different categories of smokers and drinkers (e.g. occasional, light, heavy and dependent users). Exploration of different levels of comorbid use will improve understanding of the role of cross-enforcement and cross-tolerance in the initiation and maintenance of dual drug use and the development of comorbid abuse and dependency.

In summary, alcohol and tobacco use are highly comorbid behaviours, where concurrent use may potentiate the negative effects of either substance alone. This review identified neurobiological and behavioural evidence for two central mechanisms underlying the comorbid use of alcohol and tobacco; cross-reinforcement and cross-tolerance. The findings discussed here aid our understanding of the mechanisms underlying alcohol and tobacco comorbidity and have several important implications for the comorbid treatment of alcohol and tobacco use and dependency. Development of treatment programmes should be based on the mechanisms identified in this review to improve interventions aimed at comorbidity.
